# An investigation of bedside laparoscopy in the ICU for cases of non-occlusive mesenteric ischemia

**DOI:** 10.1186/s13017-017-0118-5

**Published:** 2017-01-18

**Authors:** G. Cocorullo, A. Mirabella, N. Falco, T. Fontana, R. Tutino, L. Licari, G. Salamone, G. Scerrino, G. Gulotta

**Affiliations:** 1 General and Emergency Surgery–Policlinico P. Giaccone, University of Palermo, Via Liborio Giuffrè, 5, Palermo, Italy; 2 General and Emergency Surgery–Villa Sofia Hospita, Palermo, Italy

**Keywords:** Acute mesenteric ischemia, NOMI, Laparoscopy, Surgery, Intensive care

## Abstract

**Background:**

Acute mesenteric ischemia is a rare affection with high related mortality. NOMI presents the most important diagnostic problems and is related with the higher risk of white laparotomy. This study wants to give a contribution for the validation of laparoscopic approach in case of NOMI.

**Methods:**

Thirty-two consecutive patients were admitted in last 10 years in ICU of Paolo Giaccone University Hospital of Palermo for AMI. Diagnosis was obtained by multislice CT and selective angiography was done if clinical conditions were permissive. If necrosis was already present or suspected, surgical approach was done. Endovascular or surgical embolectomy was performed when necessary. Twenty NOMI patients underwent medical treatment performing laparoscopy 24 h later to verify the evolution of AMI. A three-port technique was used. In all patients we performed a bed side procedure 48–72 h later in both non-resected and resected group.

**Results:**

In 14 up 20 case of NOMI the disease was extended throughout the splanchnic district, in 6 patients it involved the ileum and the colon; after a first look, only 6 patients underwent resection. One patient died 35 h after diagnosis of NOMI. The second look, 48 h later, demonstrated 4 infarction recurrences in the group of resected patients and onset signs of necrosis in 5 patients of non-resected group. A total of 15 resections were performed on 11 patients. Mortality rate was 6/20–30% but it was much higher in resected group (5/11–45,5%). Non-therapeutic laparotomy was avoided in 9/20 patients and in this group mortality rate was 1/9–11%. No morbidity was recorded related to laparoscopic procedure.

**Conclusions:**

Laparoscopy could be a feasible and safety surgical approach for management of patient with NOMI. Our retrospective study demonstrates that laparoscopy don’t increase morbidity, reduce mortality avoiding non-therapeutic laparotomy.

## Background

Acute mesenteric ischemia is a rare affection with high related mortality. It accounts 1:1000 acute hospital admissions in Europe and the USA [1] and presents a very high mortality with a range from 50 to 69% [2–5] of cases.

The affection consists in an acute arterial occlusion due to embolism (EAMI), or thrombosis (TAMI), in a venous thrombosis (VAMI) or, at last, in an non-occlusive mesenteric ischemia (NOMI).

Pathophysiology is different in each type as risk factors. Different are also comorbidities and clinical findings. In all cases diagnosis is very difficult because there aren’t specific laboratory tests.

EAMI is often related to hearth disease (atrial fibrillation, myocardial infarction, etc.) and causes acute symptoms as diarrhoea, vomiting, acute abdominal pain; TAMI is characterized by more indolent onset with post-prandial pain and weight loss in patients with history of atherosclerosis, hypertension, diabetes; VAMI occurs in 10% of cases in patients with hypercoagulable disorders, malignancies, hepatitis, pancreatitis, and other affections causing slow blood flow. NOMI occurs mostly in critically ill patients with hypovolemia, hypotension, recent treatment with beta blockers or alpha adrenergic. Usually these are patients with endotracheal tube and symptoms can start in acute or gradual way.

Nowadays the gold standard for diagnosis is CT, which offers a good accuracy in AMI detection with high values of sensitivity and specificity [6], but it is well known that these values are not similar in each etiological type.

**Fig. 1:**
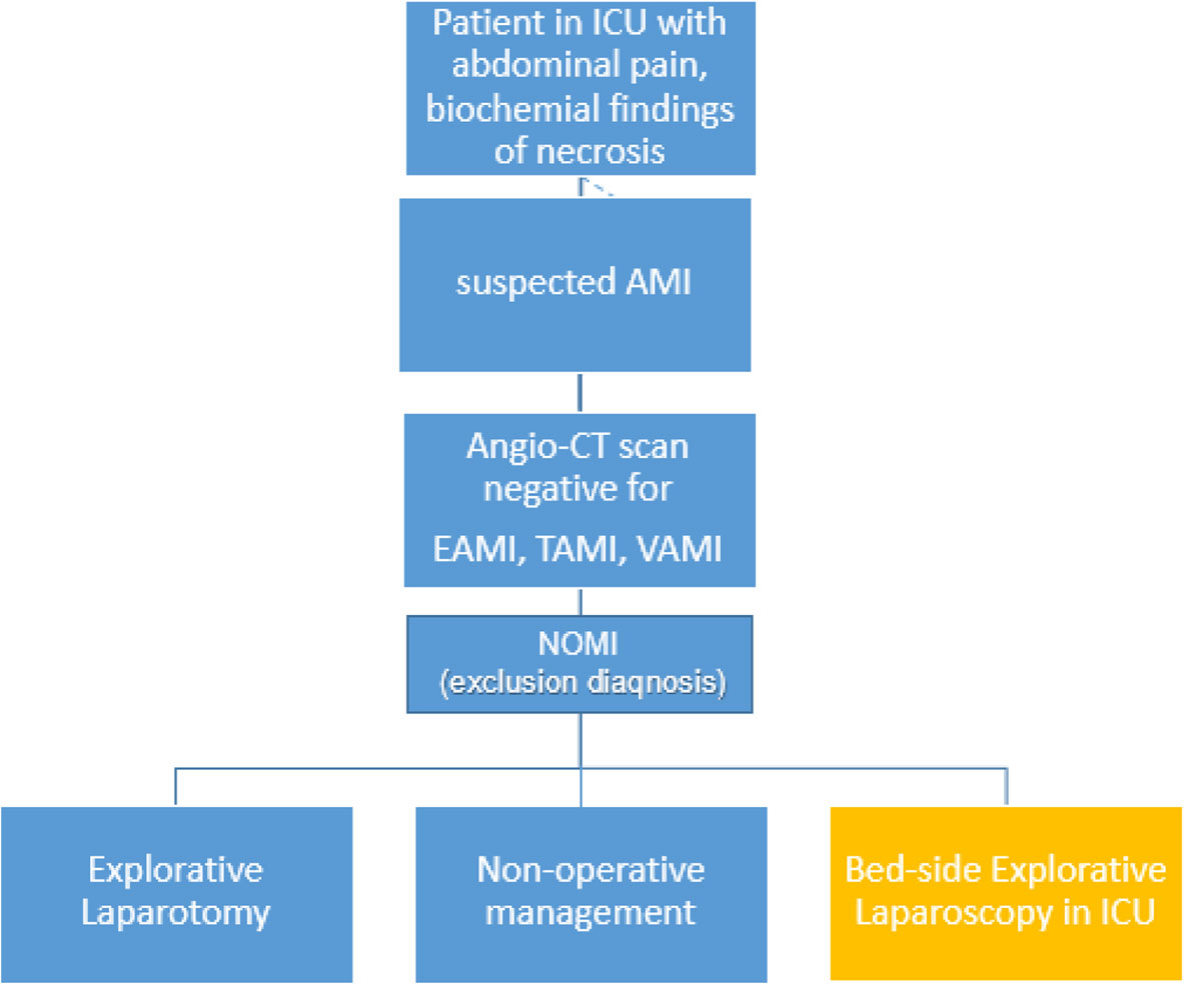
Procedural Algorithm in case of NOMI in ICU

NOMI is an exclusion diagnosis. It presents the most important diagnostic problems due to lack of specific radiological features on CT, which usually shows a normal bowel wall and a high variability of its contrast enhancement ranging from absent or diminished to increased [7]. So, in the suspicious of NOMI an anamnesis of low arterial flow or low cardiac output (recent cardiac failure, prolonged cardio-pulmonary resuscitation, cardiac surgery, severe cardiac failure, aortic dissection and aneurism, recent aortic vascular surgery etc..), biochemical findings (>TGO/>TGP;> LDL, >CPK, >Bilirubin), signs of Acute Kidney Failure (altered level of creatinine, urea and electrolytes, reduced urine output). When possible a selective angiography or an angio-CT should be performed [8] to confirm diagnosis, exclude other form of AMI and to start the medical treatment (fluid infusion, prostaglandins, etc.) (Fig. 1).

Then NOMI needs a very close follow-up to obtain an early detection of mesenteric infarction which imposes bowel resection. Early diagnosis and prompt intervention are the goals of modern treatment. It can stop the fatal progression of sepsis that is responsible of the high mortality rate [9].

Also, the treatment is different in each type of AMI [10]: resolution of embolism in open surgery (especially if bowel necrosis is present) or in endovascular way is the choice treatment in patients with EAMI or TAMI. In case of VAMI the first choice is anticoagulation and finally in patients with NOMI the first step is the infusion of fluids and vasodilators; the last mentioned are administered directly via Superior Mesenteric Artery (SMA) when possible. If bowel necrosis is present, resection is necessary at the same time [10].

Although CT consents a differential diagnosis in patients with doubtful abdominal presentation and for these reason is the first diagnostic step for these patient, there isn’t any diagnostic test which can early indicate the onset of bowel necrosis. The aim of this study is to show our results of systematic use of laparoscopy in bowel infarction detection in critical ill patients.

## Methods

A retrospective study was carried out on 32 consecutive patients recovered in last 10 years (1st January 2006–31st December 2015) in ICU of Paolo Giaccone University Hospital of Palermo. The patients’ age, clinical symptoms, biochemistry and radiological findings were considered.

**Fig 2:**
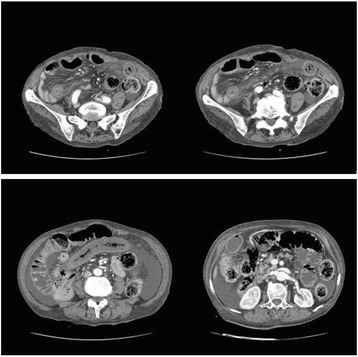
Angio-CT, a case of NOMI: Radiologic Science Department–AOUP Palermo

In all patients, AMI was diagnosed by multislice CT (Fig. 2); selective angiography was done if clinical conditions were permissive.

If necrosis was already present or suspected, surgical approach was done. Moreover, endovascular or surgical embolectomy was performed in cases with EAMI or TAMI whilst VAMI and NOMI patients underwent medical treatment performing laparoscopy 24 h later to verify the evolution of AMI.

A three-port technique was used [11, 12]: a 10-mm camera-port was positioned through the umbilical scar. After a first exploration of the abdomen, other two 5 mm operative-trocars were put in the left hypochondrium and in the left iliac fossa. In this way, as in right laparoscopic colectomy, an accurate exploration of entire small bowel was possible starting from the ileocecal junction and going back up to the Treitz ligament. Colon was entirely explored. Only in 4 patients a fourth 5 mm port in right flank was needed.

The bowel aspect and the ischemia extension were evaluated; all patients showed widespread intestinal pallor therefore, the first suffering loop was searched (intense pallor, necrosis signs) and the necrotic bowel was resected when present. The involved bowel was mobilized and after vessels ligation it was externalized through a 5–6 cm laparotomy. After resection, no anastomosis was done and an ostomy was performed. The absence of signs of necrosis is not to be underestimated because of the rapid precipitation of NOMI clinical features.

Therefore, in all patient medical therapy was continued and EBPM was administered using prophylactic dosages, the procedure was repeated 48–72 h later (Second Look) in both non-resected and resected group, looking for new necrotic areas.

Due the organization of our Hospital in nearly but separated departments, a bed-side laparoscopy was performed to avoid the transfer of the critically ill patients to the department of radiology or to operation room that often can leads to serious difficulties especially when the transfers are multiple. A laparoscopic column and a centralized CO2 distribution system are available in ICU and allow the execution of bed side laparoscopy. The availability of mobilizable beds in ICU support the surgeon to perform explorative laparoscopy with low Co2 flow and pressure (8–10 mmHg). Only two surgeons need to perform the procedure and the second or further looks are performed through the same sites used before. A 10-mm optic and two laparoscopic forceps or an ultrasound dissector allow the exploration and the dissection of bowel needs resection (Table 1). In case of re-resection the bowel was extracted trough the same previous incision and after a distal ligation of vessels, resection was performed with linear stapler. Moreover, in all cases ostomy and mucous fistula was is performed.

Safety and efficacy of the procedure was evaluated in terms of mortality, diagnosed infarctions and avoided non-therapeutic laparotomy. Postoperative morbidity was an outcome not reliable due to multiple comorbidity already present in our patients.

**Table 1:** Necessary equipment for bed-side laparoscopy

Laparoscopic Column including: CO2 insufflator, HD camera, light source, HD monitor	
Optic 10 mm	
N° 2 laparoscopic forceps	
Ultrasound dissector with disposable device	
N° 3 Trocars (10 mm, 5 mm, 5 mm)	
Surgical drapes	
Basic Surgical Kit	

## Results

Among 32 critical ill patients with CT report of AMI, 6 presented EAMI, 3 TAMI, 1 VAMI and 20 NOMI (Table 2).

**Table 2:** ICU patients with AMI

ICU patients with AMI (1st January 2006–31 December 2015)	
Type of AMI	N° of cases
EAMI	6
TAMI	3
VAMI	1
NOMI	20

Main biochemical and CT findings of NOMI patients are collected in Table 3. In all NOMI cases (20) an intense pallor of bowel wall was the main laparoscopic finding. In 14 cases, it was extended throughout the splanchnic district, whilst in 6 patients it involved mainly the ileum and the colon (right colon 2 cases; left colon 3 cases; entire colon 1 case); every patient in last group underwent resection to prevent bowel necrosis and peritonitis in 5 cases, whilst in 1 patient bowel resection was necessary to remove a necrotic segment. After a first look only 6 patients underwent bowel resection and its extension was since 15 up to 175 cm. After resection in each patient a stoma and a mucous fistula were performed on the proximal and the distal stump respectively (Table 4).

**Table 3:** Laboratory and CT findings

Patients	Age	GOT (U/L) nv: 0–31	GPT (U/L) nv: 0–31	LDH (U/L) nv: 240–480	CPK (U/L) nv: 26–192	CREATININE mg/dl nv: 0,51–0,95	WBC vn 4–11 10^3 uL	CT FINDINGS
1	66	520	489	3125	1223	5.1	26,28	negative for SMA obstruction, bowel infarction, peritoneal collections
2	79	610	498	1225	251	1,3	22,3	negative for SMA obstruction, paralytic ileum signs
3	75	426	286	1316	680	1,4	23,6	negative for SMA obstruction, paralytic ileum signs
4	54	838	778	1198	889	1,3	24,68	negative for SMA obstruction, right colon and ileum thickening
5	81	650	568	2218	1001	3,2	17,42	negative for SMA obstruction, diffuse colon and bowel infarction, peritoneal collections
6	82	466	598	1589	996	1,9	15,69	negative for SMA obstruction, paralytic ileum signs
7	61	835	687	1286	754	1,75	22,65	negative for SMA obstruction, right colon and ileum thickening
8	90	589	410	1857	1028	2,6	14,8	negative for SMA obstruction, left colon and ileum thickening
9	78	380	520	1635	987	2,4	15,1	negative for SMA obstruction, peritoneal collections
10	76	489	475	856	385	1,9	23,2	negative for SMA obstruction, bowel infarction
11	71	554	598	758	235	2,4	20,1	negative for SMA obstruction, bowel infarction, peritoneal collections
12	61	665	689	1105	624	1,4	18,7	negative for SMA obstruction, paralytic ileum signs
13	78	811	799	658	201	1,2	14,8	negative for SMA obstruction, bowel infarction
14	69	715	684	2890	1425	3,3	18,4	negative for SMA obstruction, left colon and ileum thickening
15	82	542	396	1687	1215	2,7	17,5	negative for SMA obstruction, paralytic ileum signs
16	69	496	389	1420	893	2,6	18,84	negative for SMA obstruction, bowel infarction, peritoneal collections
17	78	675	497	752	358	1,5	26,3	negative for SMA obstruction, paralytic ileum signs
18	87	742	694	3869	1845	4,8	24.3	negative for SMA obstruction, left colon and ileum thickening
19	78	868	688	1012	854	2,7	16,4	negative for SMA obstruction, paralytic ileum signs
20	72	308	258	1536	1088	3,7	37,26	negative for SMA obstruction, peritoneal collections

**Table 4:** Extension of ischemic tract in NOMI patients

Extension of Ischemia In NOMI patients		
Bowel site	N° of cases	1st look resection cases
Small Bowel and other splancnic organs	14	0
Ileum and right colon	2	2
Left colon	3	3
Entire colon	1	1

Only one non-resected patient died 35 h after diagnosis of NOMI and before the second look for cardiac failure.

The second look, 48 h later, demonstrated 4 infarction recurrences in the group of resected patients and the onset of necrosis in 5 patients of non-resected group. A total of 15 resections were performed on 11 patients (Table 5). Mortality rate was 6/20 (30%) but it was much higher in resected group (5/11–45,5%). Non-therapeutic laparotomy was avoided in 9/20 patients (45%) and in this group mortality rate was 1/9 (11,1%). No morbidity was recorded related to laparoscopic procedure (Tables 6 and 7).

**Table 5:** Recurrent necrosis after second look

Second look evaluation (48 h later)	N° of recurrent necrosis
Resected group	4
Non-resected group	5

**Table 6:** Outcome of NOMI patients after treatment

Outcome of NOMI patients after treatment	N° of cases	Resected group	Non resected group
Mortality	6/20 (30%)	5/11 (45,5%)	1/9 (11,1%)
Morbidity related to laparoscopy	0	0	0

**Table 7:** Mortality rate

Mortality	Cases/TOT	Percent
First and second look negative	1/9	11,1
1 st look positivity	3/6	50
2 nd look positivity only	2/5	40

## Discussion

NOMI is an infrequent type of AMI and accounts 20% of cases. It is more frequent in critically ill patients and depends on combination of two distinct factors; low cardiac output and vasoconstrictive agents.

In literature, there are no high evidences about clinical findings, diagnosis and therapy of AMI and even less about NOMI. It is possible to found some case-series recording the experience of single centres and in this way, the present report is a contribution about diagnostic and therapeutic pathway in critically ill patients with suspicious NOMI.

It is well known that decreased mortality for AMI in last years is related to more aggressive therapeutic approach in occlusive shapes like surgical or non-surgical blood flow restoration, resection of necrotic bowel, supportive intensive care. Moreover, the precocity of the treatment is highly related with its success.

But if in patients with occlusive forms the operative (surgical or not-surgical) approach ever follows diagnosis of AMI, in NOMI patients the treatment consists of pharmacological therapy with the need of continuous monitoring of ischemia. Only the onset of necrosis will require surgery. Because of the absence of tests that consent a determination of further bowel viability, laparoscopy can represent a diagnostic technique with high potential therapeutic options. We used it in NOMI patients both at the first and the second look to detect and remove dead bowel avoiding certain general and access-related risks associated with laparotomy [13].

Moreover, it is well known how the surgical stress could be life-threatening in these patients, and so to avoid a non-therapeutic laparotomy could be a very important step in their clinical course.

In our centre, it was started 10 years ago, routinely use of laparoscopy in critical ill patients presenting clinical and radiological findings suggesting AMI. Laparoscopy was utilized like the last diagnostic procedure and the first therapeutic step.

Explorative laparoscopy allowed to avoid 9/20 (45%) non-therapeutic laparotomies and at the same time it showed in 11 cases the presence of bowel necrosis; In 6 patients at the first look and in 9 patients at the second look. Four of second look resected patients had been already resected at the first look. The routinely execution of the second look 48 h after the first exploration of the abdomen is strongly suggested because of pathophysiology of NOMI [14]. The possible occurrence of low cardiac output due to surgical procedures (i.e. blood loss, ECC, etc.), in fact, can cause bowel ischemia but only in a variable percentage of cases necrosis will occur.

Then in our experience laparoscopy was positively used in patients with CT-scan diagnosed NOMI both for the first and the second look to detect the eventual onset of bowel necrosis. Its advantages were the possibility of bed-side performing without the surgical stress of laparotomic access.

## Conclusions

NOMI represents a frequent type of AMI diagnosis. CT scan represent the golden standard in diagnosis of AMI but has a lower power in defining NOMI forms. Laparoscopy could be a feasible and safety surgical approach for diagnosis of ischaemic tract of bowel and to removing it. Our retrospective study demonstrate that laparoscopy don’t increase morbidity and reduce mortality probably avoiding non-therapeutic laparotomy.

## Abbreviations

AMI: Acute mesenteric ischemia
CT: Computed tomography
EAMI: Embolic acute mesenteric ischemia
ICU: Intensive care unit
NOMI: Non-occlusive mesenteric ischemia
SMA: superior mesenteric artery
TAMI: Thrombotic acute mesenteric ischemia
VAMI: Venous thrombosis acute mesenteric ischemia
